# Athens Multifamily Group Therapy Project (A- MFGT) after FEP: Preliminary clinical results

**DOI:** 10.1192/j.eurpsy.2024.384

**Published:** 2024-08-27

**Authors:** M. Selakovic, A. Zartaloudi, D. Galanis, P. Valeria

**Affiliations:** ^1^Department of Psychiatry, “Sismanoglio” General Hospital; ^2^Department of Nursing, University of Western Attica; ^3^Department of Social Work, EPAPSY; ^4^First Department of Psychiatry, National & Kapodistrian University of Athens, Athens, Greece

## Abstract

**Introduction:**

The Athens Multifamily Group Therapy Project (A- MFGT) aims to provide systemic multifamily therapy to youths who experienced a first psychotic episode and their families.

**Objectives:**

Family interventions have been shown to reduce the likelihood of relapse for individuals across the spectrum of psychosis and are recommended in practice guidelines for psychosis internationally (Mc Farlane, 2016).

**Methods:**

A group of 22 young adults who presented a first psychotic episode participated with their families to multi-family group systemic therapy, after discharged from in-patient treatment. Sessions were conducted by three therapists twice a month, for nine months and supervision meetings were provided once a month. Six groups of families have been conducted since 2017. Clinical outcome was assessed through PANSS at baseline, one month later after patient’s discharge from in-patient treatment, and one year after, at the end of the multifamily group treatment. Time intervals till relapse were also assessed. Participants’ clinical findings were compared with findings from a matched group of 42 patients who did not attend the multifamily therapy program and were treated as usual.

**Results:**

Two-way mixed ANOVA was conducted to assess PANSS scores change over time (t1: at base line, t2: at one month and t3: one year), while differences were investigated between the two groups of patients and interactions were checked. Regarding PANSS-positive scale and PANSS-general scale, no differences were found between the two groups in neither of the three time points. Regarding PANSS-negative scale, patients attending MFGT presented statistically significantly lower scores in t3 than patients treated as usual, but not in t1 and in t2 (i.e., prior to therapy). Moreover, both patients’ group showed improvement from t1 to t2, but only patients attending MFGT further improved from t2 to t3. Among patients attending MFGT, two (9.1%) had a relapse compared to nine (22.5%) of the patients treated as usual, however this comparison did not reach statistical significance (p = 0.300).

**Conclusions:**

In term to provide early intervention in psychosis, A-MFGT seems to be a viable way to support the patient as well as the whole system facing psychosis, with the aim of preventing relapse and improved quality of life for all the participants.

**Disclosure of Interest:**

None Declared

